# Promoters Architecture-Based Mechanism for Noise-Induced Oscillations in a Single-Gene Circuit

**DOI:** 10.1371/journal.pone.0151086

**Published:** 2016-03-09

**Authors:** N. Guisoni, D. Monteoliva, L. Diambra

**Affiliations:** 1 Instituto de Física de Líquidos y Sistemas Biológicos, Universidad Nacional de La Plata, La Plata, Argentina; 2 Departamento de Física, Facultad de Ciencias Exactas, Universidad Nacional de La Plata, La Plata, Argentina; 3 Centro Regional de Estudios Genómicos, Universidad Nacional de La Plata, La Plata, Argentina; University of Edinburgh, UNITED KINGDOM

## Abstract

It is well known that single-gene circuits with negative feedback loop can lead to oscillatory gene expression when they operate with time delay. In order to generate these oscillations many processes can contribute to properly timing such delay. Here we show that the time delay coming from the transitions between internal states of the *cis*-regulatory system (CRS) can drive sustained oscillations in an auto-repressive single-gene circuit operating in a small volume like a cell. We found that the cooperative binding of repressor molecules is not mandatory for a oscillatory behavior if there are enough binding sites in the CRS. These oscillations depend on an adequate balance between the CRS kinetic, and the synthesis/degradation rates of repressor molecules. This finding suggest that the multi-site CRS architecture can play a key role for oscillatory behavior of gene expression. Finally, our results can also help to synthetic biologists on the design of the promoters architecture for new genetic oscillatory circuits.

## Introduction

Oscillatory phenomena are an essential feature of biological systems and such behavior is present at different levels of the organization of the living matter (cell, tissues, organs and individuals). At the intra-cellular level several examples of genes with oscillatory expression are known, whose periods range from ∼ 40 minutes in the zebrafish somitogeneses [1] to a day in circadian clocks [2]. In general, the mechanism underlying such oscillations is a negative regulatory loop implemented in a gene-protein interaction network. The complexity of such networks vary from highly complex ones, as those described for the cell division cycle, or the circadian rhythm, to the simplest ones which were synthetically implemented in prokaryotic cells [3, 4]. In this sense Stricker *et al*. have shown that a synthetic single-gene circuit is able to display oscillatory behavior [5]. It is well known that time delay serves as a source of instabilities which can lead to oscillations and also to chaotic behavior [6, 7]. The dynamical system theory predicts that single-gene circuits with negative feedback loop can exhibit oscillatory gene expression when they operate with an explicit time delay [8–11], or when such time delay is implicit in additional steps representing post-transcriptional events [12, 13]. Several processes, such as: transcript elongation, splicing, translocation, translation and phosphorylation, can contribute to generate a proper time delay [14]. The resulting period of the oscillation emerges from the combination of such time consuming processes rather than the sum of the individual delays, because such processes can occur concurrently. Recently, researchers have assessed experimentally the contribution of processes such as transcript elongation [15], splice processing [16], and nuclear translocation [17] on the oscillatory behavior. However, the individual impact of such processes on the dynamics of the circuit is not yet fully understood, offering to modelers an opportunity to assess them theoretically. In this paper we show an alternative mechanism able to provide the time delay needed for generate sustained oscillation. This mechanism is based on transitions between internal states of the CRS rather than in post-transcriptional events. To examine if this mechanism can generate sustainable oscillations *per se*, we devise an autorepressive single-gene loop without explicit time delay nor additional post-transcriptional events. Our analysis show that the proposed mechanism is able to generate oscillations when operates in a stochastic regime, but not in a deterministic scenario where the system exhibits a fixed point. This result suggests that the CRS architecture can constitute a mechanism for noise-induced oscillations (NIO). NIO phenomenon was first reported associated with stable focus by McKane and Newman [18]. NIO was also reported in a chain of downstream-coupled Brusselators [19]. Furthermore, Toner *et al*. have recently shown that intrinsic chemical fluctuations can also induce concentration oscillations in systems whose deterministic models exhibit a stable node [20].

## Materials and Methods

### The model

**Fig 1:**
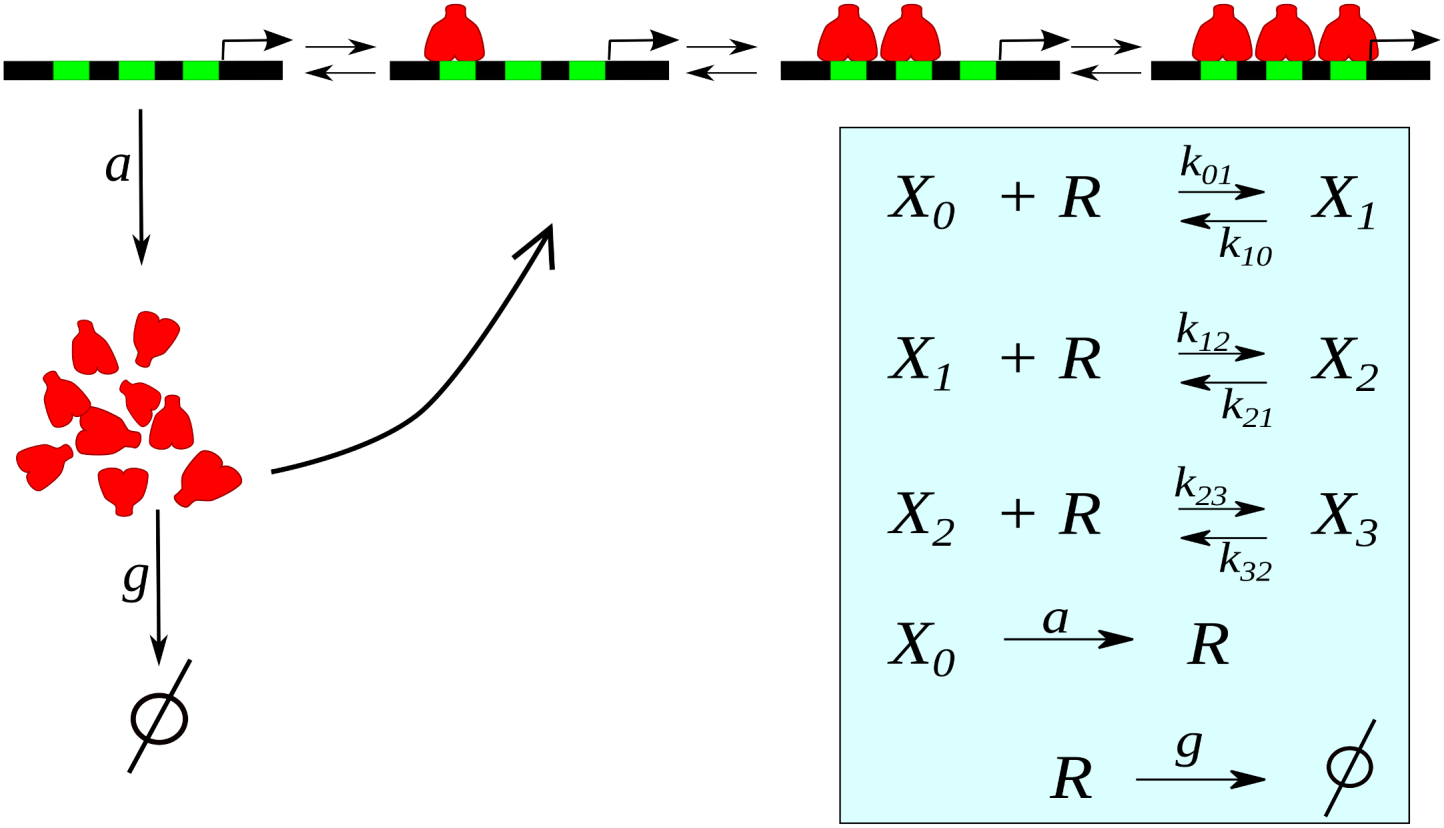
Sketch of the autorepressive single-gene loop with three binding sites. The repressor molecules *R* (red) can bind to regulatory sites (green) on the DNA inhibiting its own synthesis. Inset: Cascade of reactions where *X*_*i*_ represents the promoter with *i* bound repressors, and *k*_*i*,*j*_ the transition rates.

Our model considers that gene expression is regulated by a tandem of *N* functionally identical regulatory binding sites, where the gene product can bind cooperatively inhibiting its own expression (see Fig 1). This architecture has a biological counterpart in the mouse Hes1 and Hes7 genes (associated to the somitogenic clocks of mammalians), which negatively autoregulate its own expression through three binding sites (an N- and two E-boxes sequences) in the proximal promoter [21]. In order to emphasize the role of *cis*-regulatory system dynamics as an alternative oscillatory mechanism, we do not take into account the translation step. Also, for simplicity, we consider that the repressor synthesis occurs, at rate *a*, only when no repressor is bound to the DNA, and that they are linearly degraded at rate *g*. In fact, the present model is a modified version of a previous one with many binding sites [22, 23], by including a negative feedback loop.

### The macroscopic description

The deterministic reaction rate equations for the proposed model can be written as

$$\begin{array}{c} \frac{d\vec{\phi }}{dt}=S\vec{f}(\vec{\phi }), \end{array}$$ (1)

where $\vec{\phi }$ is the vector of concentrations, **S** is the stoichiometric matrix, and $\vec{f}$ is a vector function whose component *f*_*j*_ is the rate function of the *j*-th reaction. In our case *ϕ*_*i*_ is the fraction of genes with *i* = 0, 1, …, *N* bound molecules, and *ϕ*_*N*+1_ is the concentration of repressors *R*, which will be denoted hereafter by *c*. For the example of Fig 1, when *N* = 3, the system comprises five chemical species and eight first-order chemical reactions. The stoichiometric matrix is given by:

$$\begin{array}{c} S=(\begin{array}{cccccccc} -1 & 1 & 0 & 0 & 0 & 0 & 0 & 0\\ 1 & -1 & -1 & 1 & 0 & 0 & 0 & 0\\ 0 & 0 & 1 & -1 & -1 & 1 & 0 & 0\\ 0 & 0 & 0 & 0 & 1 & -1 & 0 & 0\\ -1 & 1 & -1 & 1 & -1 & 1 & 1 & -1 \end{array}), \end{array}$$

and the corresponding deterministic reaction rate equations are:

$$\begin{array}{ccc} \frac{d\phi_{0}}{dt} & = & -k_{01}\phi_{0}c+k_{10}\phi_{1},\\ \frac{d\phi_{1}}{dt} & = & -k_{12}\phi_{1}c-k_{10}\phi_{1}+k_{01}\phi_{0}c+k_{21}\phi_{2},\\ \frac{d\phi_{2}}{dt} & = & -k_{23}\phi_{2}c-k_{21}\phi_{2}+k_{12}\phi_{1}c+k_{32}\phi_{3},\\ \frac{d\phi_{3}}{dt} & = & -k_{32}\phi_{3}+k_{23}\phi_{2}c,\\ \frac{dc}{dt} & = & a\phi_{0}-gc+k_{10}\phi_{1}+k_{21}\phi_{2}+k_{32}\phi_{3}-c\left(k_{01}\phi_{0}+k_{12}\phi_{1}+k_{23}\phi_{2}\right), \end{array}$$ (2)

where *k*_*ij*_ is the kinetic rate between promoter states *i* and *j*. Due to cooperativity, previous binding of a repressor molecule alters the actual binding or unbinding process. Known relationships between the system’s kinetics and thermodynamic properties allow us to write all kinetic rates, *k*_*s*,*s*+1_ and *k*_*s*+1, *s*_ in terms of three parameters [22]: the binding rate *p*, the unbinding rate *q*, and *ϵ* which represents the cooperativity intensity, i.e., ($\epsilon =e^{-\frac{\Delta G_{I}}{RT}}$, where $\Delta G_{I}$ is the free energy of the interaction). We consider that the presence of already bound repressors alter DNA affinities increasing binding rates *k*_*s*,*s*+1_. Thus, following [22], we can write *k*_*s*,*s*+1_ = *ϵ*^*s*−1^(*N* + 1 − *s*)*p*, while *k*_*s*+1, *s*_ = *s* *q*. Of course *ϵ* = 1 indicate the absence of cooperativity. An analytic exploration by standard linear analysis around the steady state of this system is only possible for *N* ≤ 2. However, if the synthesis and degradation processes are much slower than the CRS kinetics, we can perform a quasi-steady state approximation on *ϕ*_*i*_ variables, with *i* = 0, …, *N*, which allows to write the temporal evolution of the repressors concentration as $\dot{c}=a\;F\left(c\right)-g\;c$. *F*(*c*) is a sigmoidal regulatory function with an effective half-maximum concentration *K*_*d*_, and effective steepness *n*_*H*_. Such simplified system does not present limit cycle independently on the steepness of the monotonically decreasing regulatory function *F*.

### The mesoscopic description

The macroscopic Eq (1) may be not suitable to describe correctly the gene expression regulation due to the low copy numbers of the involved chemical species. This lead to the necessity of a stochastic description of the system. To this end we consider stochastic simulations of the elementary reactions [24], and the strategy based on the linear-noise approximation (LNA). The LNA is broadly used to study how noise affects reaction networks at the cellular level [25–27]. This approach provides exact predictions for reaction networks composed of zero- and first-order reactions, or for networks involving second-order reactions and large volumes at constant concentrations. Recently, it has been proved that the precision of LNA predictions can be extended to a strict class of chemical systems with second-order reactions for all volumes [28]. This approach has been previously used to estimate power spectrum derived from biochemical circuits [20, 27], and from genetic oscillators [26, 29]. In this approximation the fluctuations $\vec{\eta }$ around the mean concentrations Eq (1) are given by [25]:

$$\begin{array}{c} d\vec{\eta }=J\vec{\eta }\left(t\right)dt+Bd\vec{W}\left(t\right), \end{array}$$ (3)

where J and $D={\text{BB}}^{T}$ are the Jacobian and diffusion matrices, respectively, and $d\vec{W}$ is a Wiener process. Matrices J and D can be computed from the rate equations and from the stoichiometry matrix as $J_{ij}=\frac{\partial (S_{i}\cdot \vec{f}(\vec{\phi }))}{\partial \phi_{j}},$ and $D=S\;\text{diag}\left(\vec{f}\right)\;S^{T}$ [25]. This approximation allow us to write the autocorrelation function 〈*η*_*i*_(*t*)*η*_*i*_(*t*+*τ*)〉, in terms of the J and D matrices, from which one can estimate the power spectrum.

### The power spectrum

In order to assess the oscillatory behavior of the model above, we are interested in compute the power spectrum of the fluctuations associated with the number of transcripts. This power spectrum is computed in two ways. The first one is from the time series derived from stochastic simulations. In this case, the spectrum is calculated by averaging the 8000 periodograms from realizations of 820 min length, and then normalizing by the total power. As usual the periodogram was computed using the discrete Fourier transform [30].

As this estimation are computational expensive to explore the parameter space, we have also used as a second alternative, a closed-form equation for the power spectrum of fluctuations derived from the autocorrelation function, in the LNA framework [20]. Thus, the power spectrum of fluctuations associated to *i*–esima specie is given by:

$$\begin{array}{c} S_{i}\left(\omega \right)=\frac{\Omega }{\pi }{\left[{\left(-J+I\;i\omega \right)}^{-1}D{\left(-J^{T}-I\;i\omega \right)}^{-1}\right]}_{ii}, \end{array}$$ (4)

where Ω is the volume, and I is the identity matrix. Once determined the spectrum, one important issue in the study of stochastic oscillations is to distinguish them from the random fluctuations using the peaks of the spectrum. Therefore, we will consider an oscillation quality measure [20], $Q_{90\%}=\hat{\omega }/\Delta \omega$, where $\hat{\omega }$ is the peak frequency of NIO, and Δ*ω* is the difference between the two frequencies at which the power takes its 90% of the peak value. We choose this formalism for its simplicity, despite of the limitations of its predictions when one is dealing with low copy numbers of genes, as our case. In this sense, it is important to remark two limitations of the LNA for our purpose: (i) it can significantly underestimate the period and amplitude of the oscillations [26], and (ii) it is able to predict only the main peak of the spectrum [31]. A more general discussion about the limitations of the LNA for chemical systems can be found in [32, 33].

## Results

**Fig 2:**
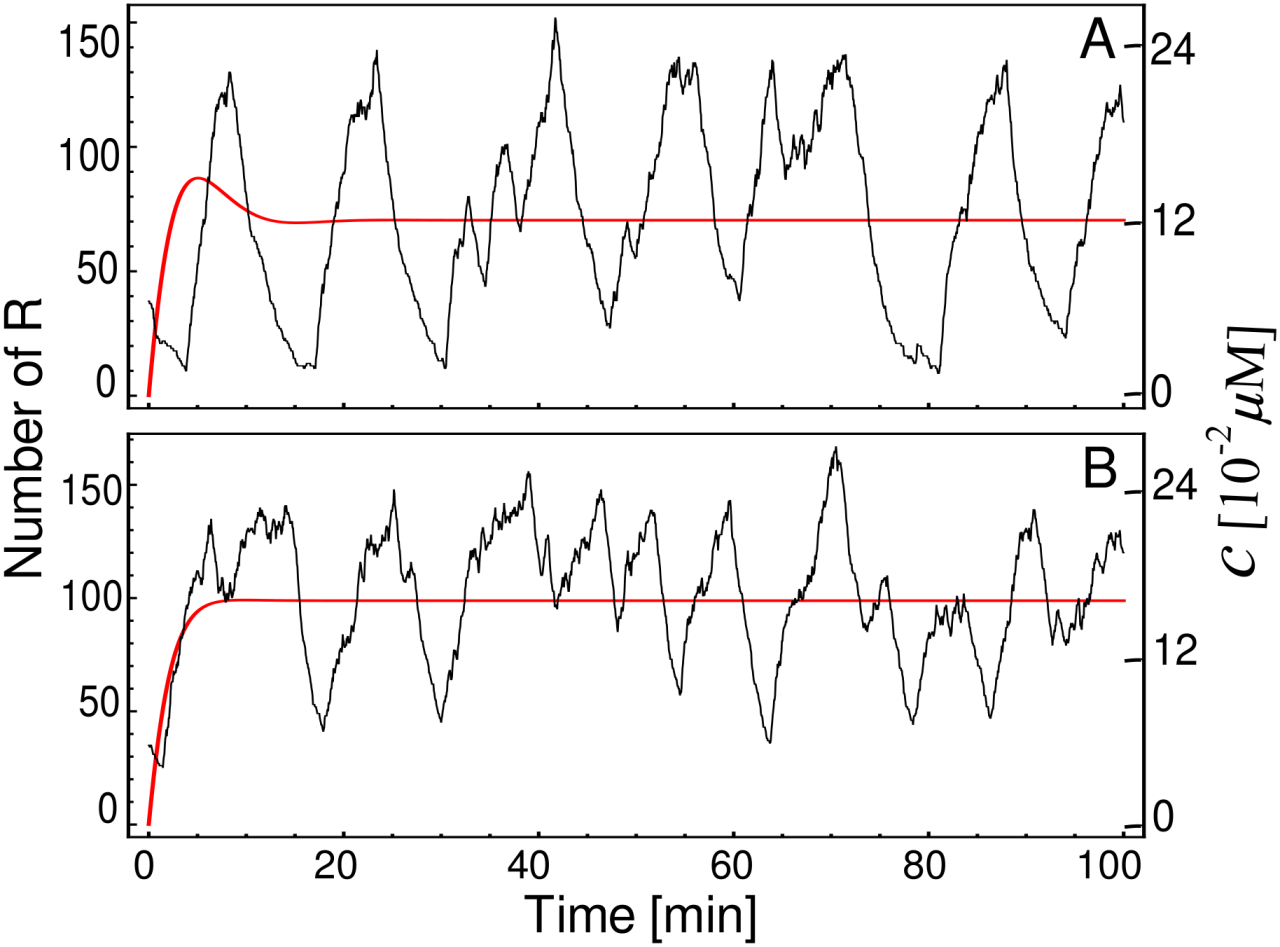
Deterministic vs. stochastic descriptive levels. Temporal course of the concentration *c* (red lines), obtained by numerical integration of Eq (1), and stochastic trajectories (black lines) of the same system displaying an oscillatory behavior. The parameter values are *N* = 3, *p* = 1.7 × 10^−3^ *μ*M min^−1^, *q* = 0.75 min^−1^, *a* = 0.075 *μ*M min^−1^, and *g* = 0.3 min^−1^. In addition *ϵ* takes value of 9 for case (A) and 2 for case (B). For stochastic simulations we consider the cell volume as Ω = 1 × 10^−15^ *l*.

To verify if a mechanism based on transitions between internal states of the CRS can generate sustainable oscillations *per se*, we perform simulations of the model sketched in Fig 1 at two different descriptive levels. A macroscopic description can be obtained by numerical integration of Eq (1). In this case, the system presents a damped oscillation before reaching the fixed point (Fig 2A, red line) for *N* = 3, *p* = 1.7 × 10^−3^ (*μ*M min)^−1^, *q* = 0.75 min^−1^, *ϵ* = 9, *a* = 0.075 *μ*M min^−1^, and *g* = 0.3 min^−1^ (see parameters values in [8, 22], and references therein). On the other hand, an exact stochastic simulation of these same reactions exhibits oscillations (Fig 2A, black lines) similar to the degrade-and-fire oscillations reported in [34] which uses an explicit time delay model. Oscillations in deterministic single-gene circuits without delay have been described in Goodwin-like models [14] which differ from the presented here because they consider two additional post-transcriptional steps. However, the oscillatory behavior in the Goodwin model occurs for *n*_*H*_ > 8 [12, 35]. This high value of the Hill coefficient at the transcriptional level has been considered rather unrealistic [14]. The prediction of such high *n*_*H*_-value could be explained as a consequence of the quasi-steady state approximation underlying to such models and to an inadequate macroscopic-deterministic description. For the case of a lower cooperativity (Fig 2B, *ϵ* = 2), where the deterministic system does not exhibit damped oscillations, the oscillatory-like behavior of the stochastic counterpart is less apparent and more difficult to be distinguished from noise, as expected for lower cooperativity. The results above show that the transitions between internal states of the CRS can constitute a mechanism for noise-induced oscillations in gene expression. Consequently, the CRS architecture can play an important role on oscillatory genetic circuits.

**Fig 3:**
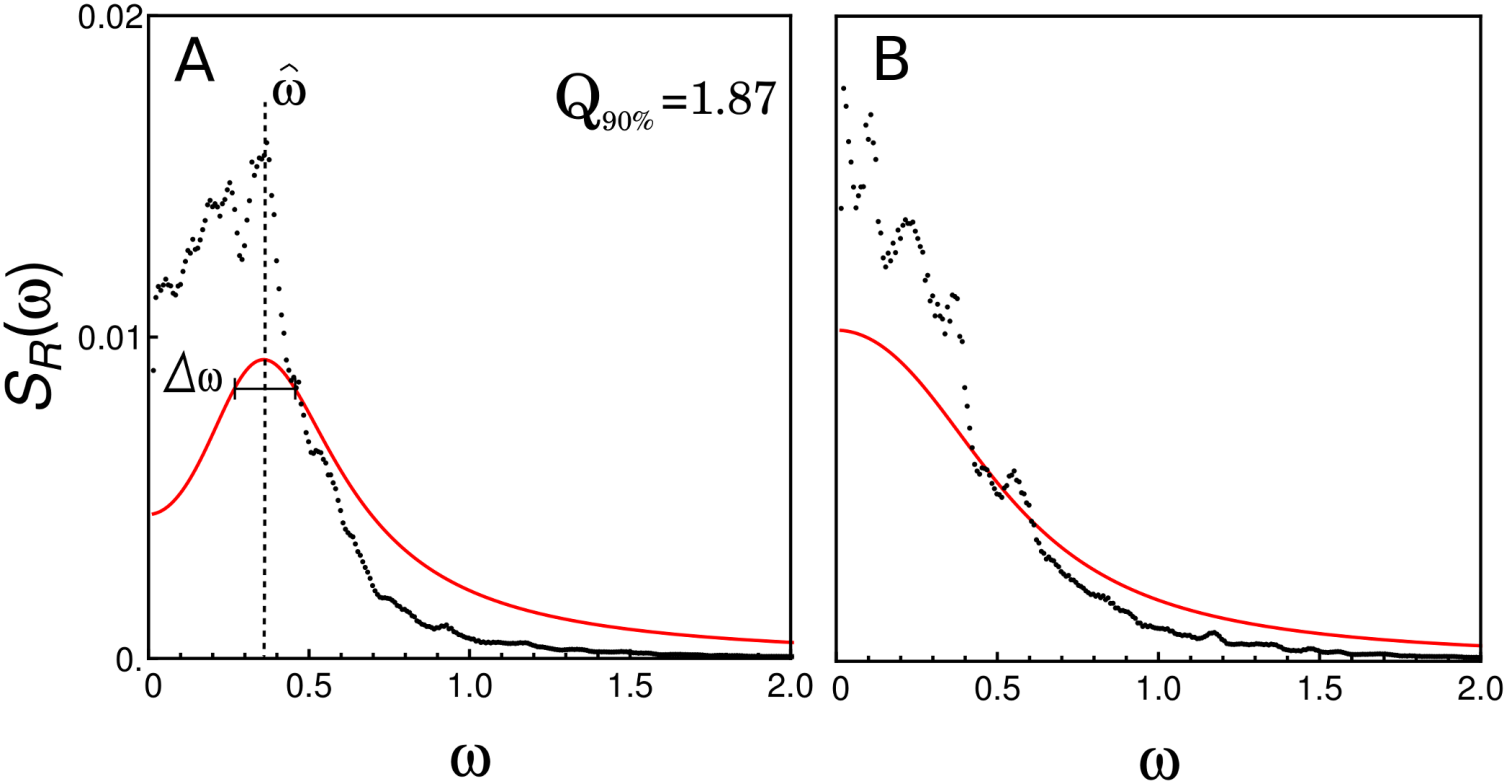
Power spectral densities. Normalized power spectral density of the fluctuations of repressor obtained by averaging 8000 periodograms from stochastic simulations of Fig 2 (black dots), and the approximate normalized power spectral density computed by using Eq (4) (red curve). $\hat{\omega }$ indicates the peak frequency, and Δ*ω* is the difference of the two frequencies at which the power takes the 90% of the peak value. The frequency *ω* is given in radians per minute (rad min^−1^).

Fig 3 shows the normalized numerical spectrum (black dots) estimated from the exact stochastic simulations displayed in Fig 2 and, for comparison, the corresponding power spectrum computed by Eq (4) (red curve). The numerical spectrum show peaks, which are more evident in the case of *ϵ* = 9 (Fig 3A), where there is a stable focus. On the other hand, the approximated power spectral densities exhibit only one peak, at *ω* = 0.37, in the case of *ϵ* = 9, in good agreement with the power spectrum computed from stochastic simulations. However, the LNA does not reflect the small peaks that also appear with *ϵ* = 2 (Fig 3B). The observed discrepancies between the numerical and the approximate spectrum are due to the limitations of LNA to describe systems with very low copy number of genes, and due to the high nonlinearity because of the feedback loop. In the cases of Fig 3, the quality factor for the sustained oscillation case (Fig 3A, *ϵ* = 9) is *Q*_90%_ = 1.87, while it is not defined for the case with lower cooperativity (Fig 3B, *ϵ* = 2). Increasing *p*-value 100-fold in case of *ϵ* = 9 also leads to oscillations (data not shown). The periods of the oscillations in Fig 3A (∼15 min) are smaller when contrasted with the fastest oscillations in eukaryotes, however by including other time-consuming steps in the model, as elongation, protein synthesis and translocation, the oscillation period could increase significatively.

Beyond showing evidence on NIO phenomenon in a single-gene circuit without explicit delay, we want to study two key features. The first one is the steepness of the regulatory function, which is determined both by the intensity of the cooperative binding *ϵ*, and the number of regulatory binding sites *N*. The second one is the interplay between the CRS kinetic and the rate of synthesis and degradation processes. To unshroud the influence of this relationship, we scaled the rates of the synthesis and degradation processes with a parameter *λ*, writing *a* = *λa*_0_, and *g* = *λg*_0_. Thus, increasing *λ* speeds up the synthesis/degradation processes in relation to the CRS, but without altering the mean number of repressor molecules nor the resulting regulatory function.

**Fig 4:**
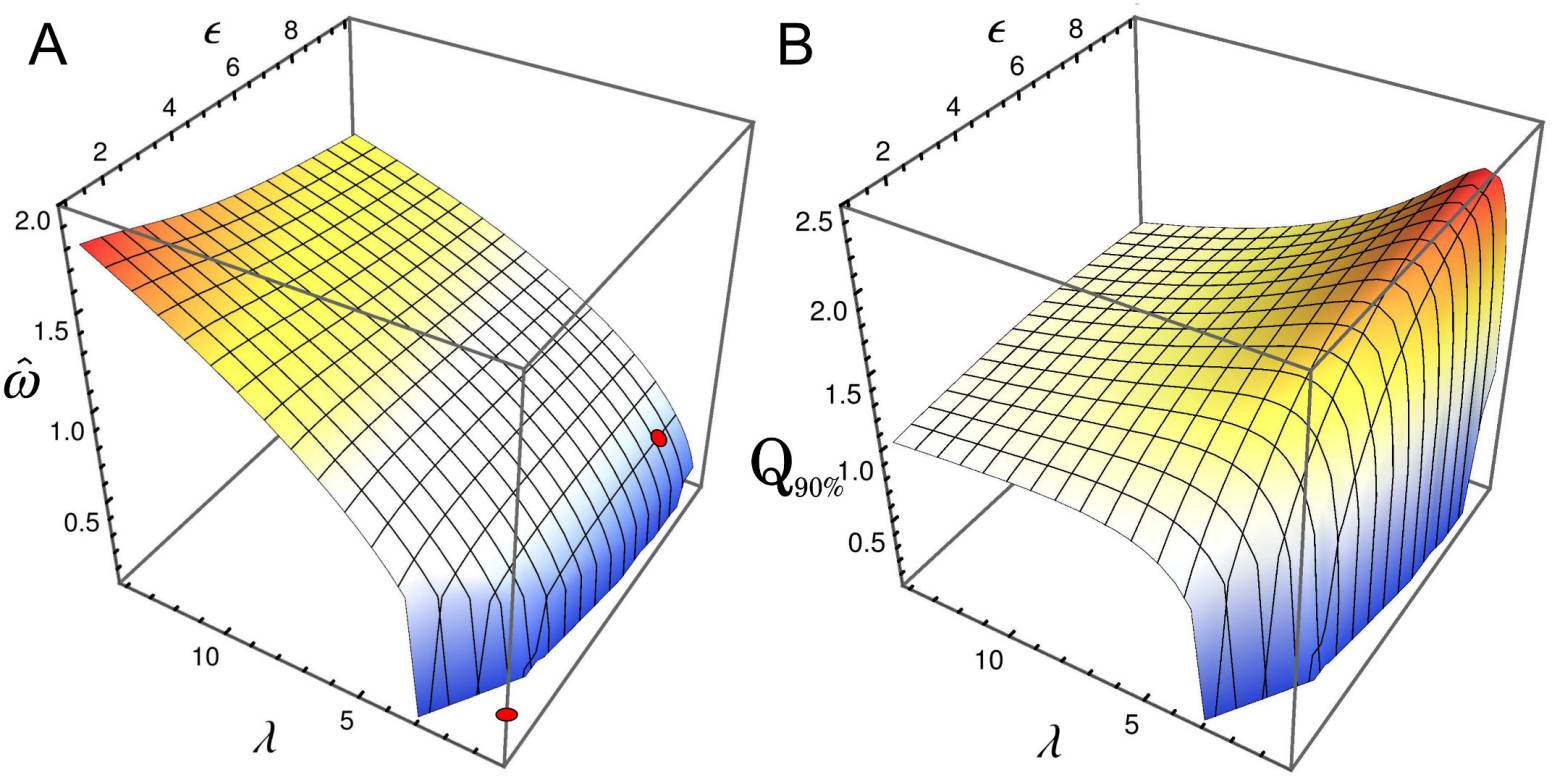
Influence of kinetic rates and cooperativity on oscillations. Peak frequency $\hat{\omega }$ (panel A) and the quality factor *Q*_90%_ (panel B) as a function of *ϵ* and *λ*, for oscillations in the number fluctuations of repressor. Red dots correspond to the parameter values used in Figs 2 and 3, where *λ* = 1. See details of the plot near *λ* = 1 in S1 Fig.

Fig 4A and 4B depict the peak frequency $\hat{\omega }$ and the corresponding quality factor *Q*_90%_, respectively, of the oscillatory behavior as a function of *ϵ* and *λ* for the oscillations in the number fluctuations of repressor. For the parameter values studied here (the same as in Fig 2 with *a*_0_ = 0.075 *μ*M min^−1^ and *g*_0_ = 0.3 min^−1^) the oscillation period ranges from 3 to 15 minutes, reaching the maximum frequency at high values of lambda, i.e. high rates of the synthesis/degradation processes (left panel). However, in this regime the quality of oscillations is poor, as one can observe in the right panel. The *Q*_90%_-factor shows that there is a particular range for the synthesis/degradation rates, around *λ* = 2, where the NIO become particularly evident. Fig 4B also shows that the auto-regulatory circuit proposed here can not present clear oscillatory behavior for slow rates of synthesis/degradation processes (*λ* < 2). One also can observe from Fig 4B that increasing *ϵ* improves the NIO phenomena.

**Fig 5:**
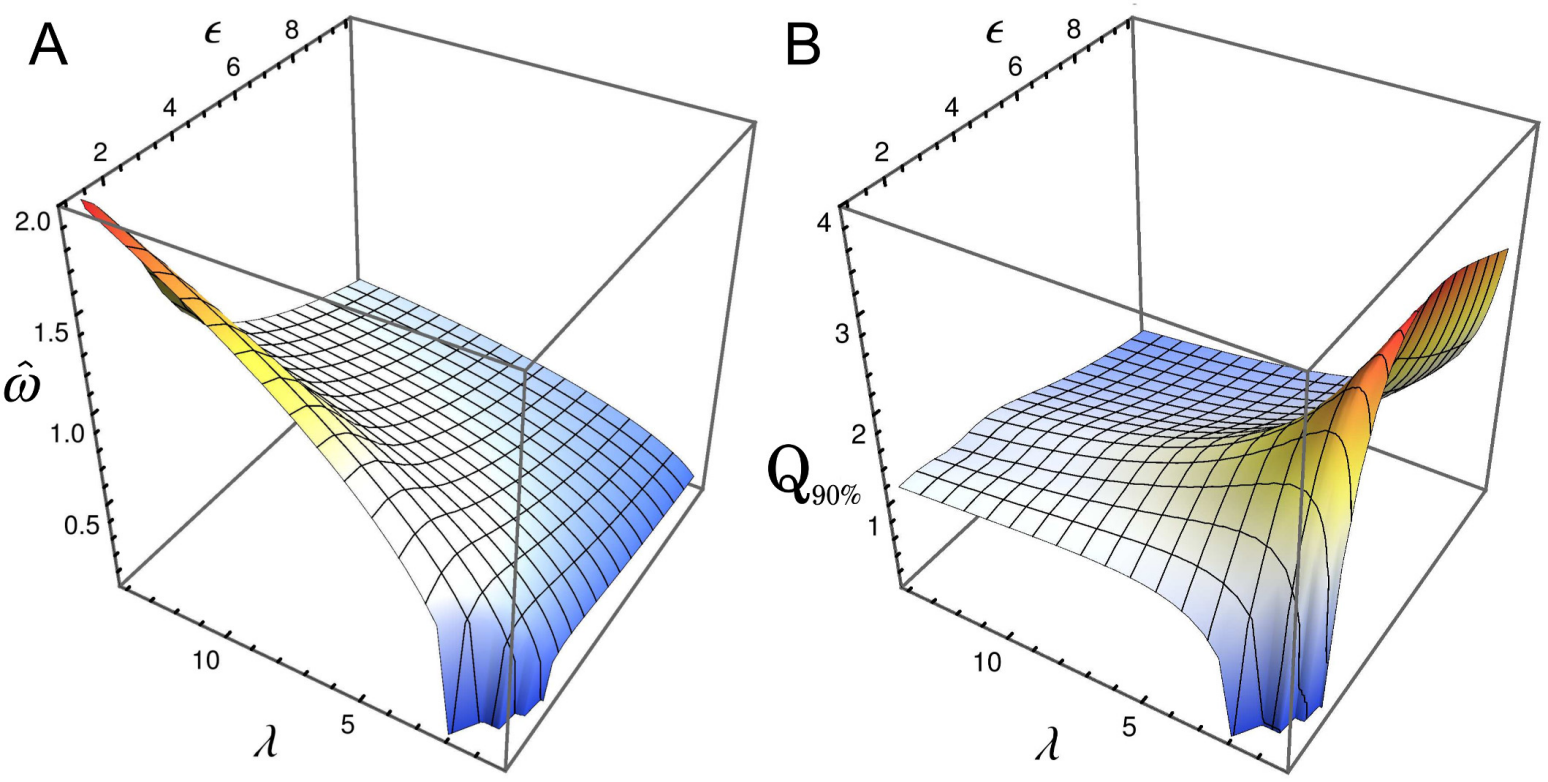
The number of binding sites enhances oscillatory behavior. Peak frequency $\hat{\omega }$ (panel A) and the quality factor *Q*_90%_ (panel B) as a function of *ϵ* and *λ*, for oscillations in the number fluctuations of repressor for the same parameter than Fig 4, but with *N* = 5 instead of *N* = 3.

We have also studied what happens when we increase the steepness of the effective regulatory function by increasing the number of binding sites, keeping constant all other parameters. For the case of *N* = 5, one observes a slight decrease of the peak frequency when increasing *ϵ*, as shown in Fig 5. On the other hand, as expected, the *Q*_90%_-factor increases markedly when the number of regulatory binding sites increases, as can be seen in Fig 5B. In this sense, for *λ* = 1 and moderate *ϵ* values the *Q*_90%_-factor reaches higher values than in Fig 3B. An interesting feature shown in Fig 5B is that *Q*_90%_-factor does not increase monotonically with *ϵ*. This behavior can be linked with the fact that fluctuation levels, and probably the width of the peak frequency, increases with *ϵ* [23].

**Fig 6:**
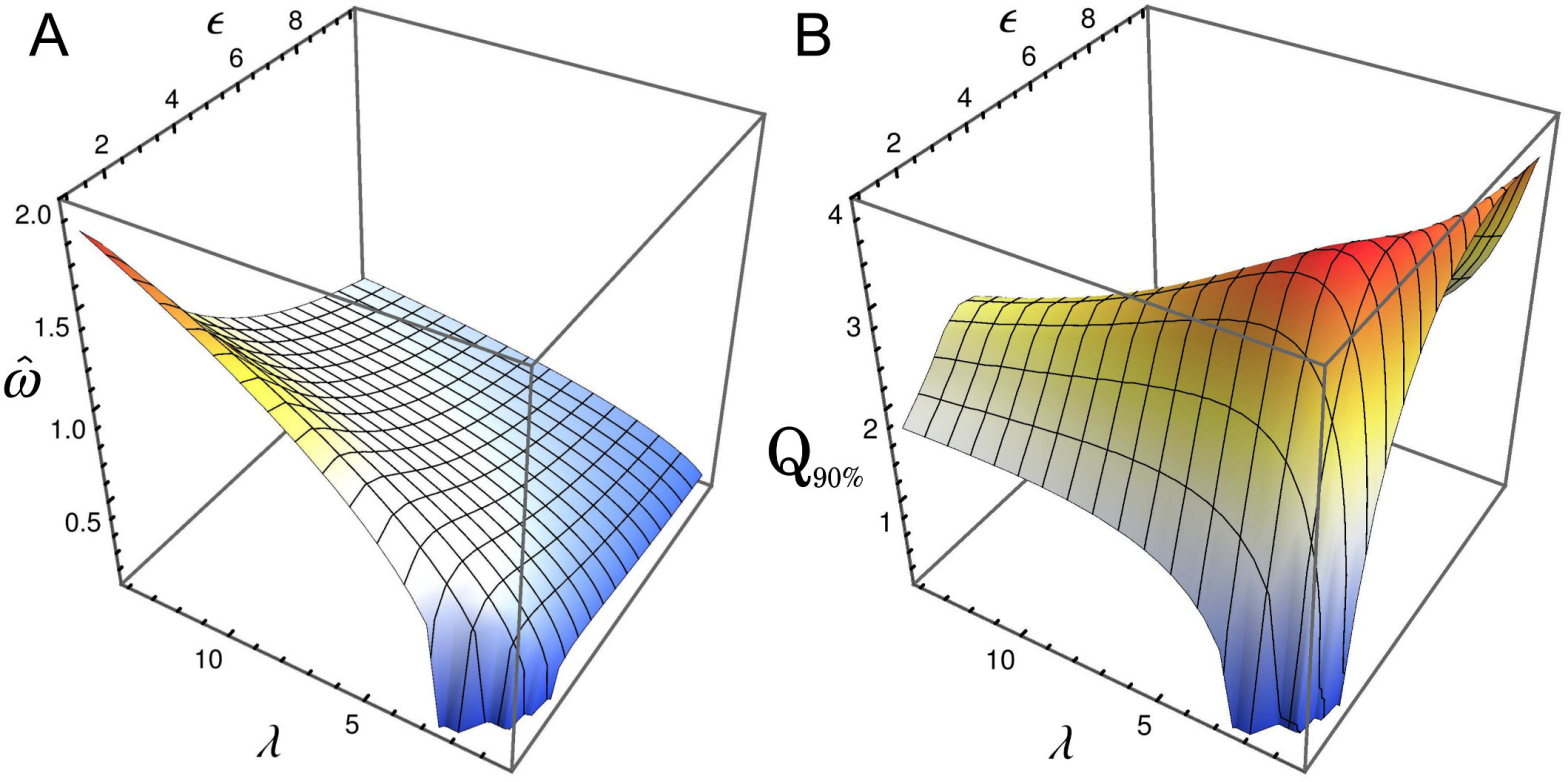
Improving oscillations by increasing the occupancy of all binding sites. Peak frequency $\hat{\omega }$ (panel A) and the quality factor *Q*_90%_ (panel B) as a function of *ϵ* and *λ*, for oscillations in the number fluctuations of repressor for the same parameter as Fig 5, but with *a*_0_ = 0.15 *μ*M min^−1^ and *g*_0_ = 0.15 min^−1^.

As illustrated by the previous examples, autorepressive single-gene circuit with multi-site CRS can exhibit oscillatory behavior in a mesoscopic regime without the necessity of explicit time lagged variables, or post-transcriptional step. The oscillatory behavior illustrated above can be explained in terms of transitions between the internal states of the CRS, which are able to buffer time in the same way as additional post-transcripcional steps. In our model the time-buffering capability is determined by the mean number of sites occupied when there is a high number of repressor molecules. Consequently, for a given CRS kinetics the expression level of repressors that reach the system could be an important parameter for NIO development. To illustrate this idea we consider the same parameters for CRS as in Fig 5, but increasing *a*_0_ and decreasing *g*_0_ parameters by the same factor (*a*_0_ = 0.15 *μ*M min^−1^ and *g*_0_ = 0.15 min^−1^), in order to increase the expression level. Fig 6 shows that the peak frequency $\hat{\omega }$ is almost the same as Fig 5A, while the quality factor *Q*_90%_ gives clear evidence that the oscillatory behavior becomes much more apparent. Remarkably, in this regime it is possible to find good oscillations even in the absence of cooperative binding (i.e., *ϵ* = 1).

## Discussion

In summary, we have presented a model that considers a multi-site CRS architecture and allows to unravel the role of different elements that conform the circuit (binding sites, cooperative interactions, kinetic rates) in the development of sustained oscillatory behavior. Our stochastic analysis reveals that oscillations in the expression level of repressor are feasible in a broad range of parameters, while the corresponding macroscopic reaction rate equations predict the existence of stable fixed points. To the best of our knowledge, the existence of phenomena NIO has previously been predicted solely in genetic oscillators with explicit time delay [36, 37].

We find that, the steepness of the regulatory function *F* plays a key role in NIO development. In this sense, both the intensity of the cooperative interaction between transcription factors and the number of regulatory binding sites on the CRS can enhance the oscillatory behavior. However, the cooperative interaction is not an essential ingredient because we found oscillatory behavior even in a total absence of cooperativity. Not trivial behavior in absence of cooperativity was also reported in genetic toggle switch [38, 39]. This fact is in contrast with a recent finding about instabilities on spatially extended systems, where cooperative binding is essential for Turing patterns arising [40]. On the other hand, recent studies have been shown that Turing patterns can also be promoted by chemical noise [41, 42], in an equivalent manner to the NIO phenomenon. Furthermore, we show that by increasing the expression level of the system improves the quality of the sustained oscillations. This effect is derived from the multi-site CRS architecture, because at higher levels of repressor, the system have more chance to occupy all binding sites during the resting phase, which in turn increases the implicit delay [43].

Our finding emphasize the role of the multi-site CRS architecture for naturally occurring genetic oscillators, such as genes Hes1 and Hes7. The expression of these genes is inhibited by their own products which can bind to three regulatory binding sites [1], as proposed here. We have shown that in a multi-site promoter the activation/deactivation process contributes to the overall time delay and can drive oscillations *per se*. However, though the periods of the oscillations reported here are smaller than the period of the segmentation clocks, we expect that longer periods can be reached by including in the model other time-consuming processes [15–17].

We believe that the use of detailed, and biologically interpretable, CRS model in combination with stochastic analyses, offers new insights into the nature of these oscillations, especially in the context of segmentation clocks, as well as potentially aiding in the design of new synthetic biological prototypes.

## Supporting Information

S1 FigDetails of Fig 4.Peak frequency $\hat{\omega }$ (top panel) and the quality factor *Q*_90%_ (bottom panel) as a function of *ϵ*, for three value of *λ*: 0.75 (red), 1.0 (green) and 1.25 (blue).(TIF)
