# Supplementary figures and images for: Promoters Architecture-Based Mechanism for Noise-Induced Oscillations in a Single-Gene Circuit
Source: PLoS One. 2016 Mar 9;11(3):e0151086. doi: 10.1371/journal.pone.0151086 (PMC4784906; doi:10.1371/journal.pone.0151086)

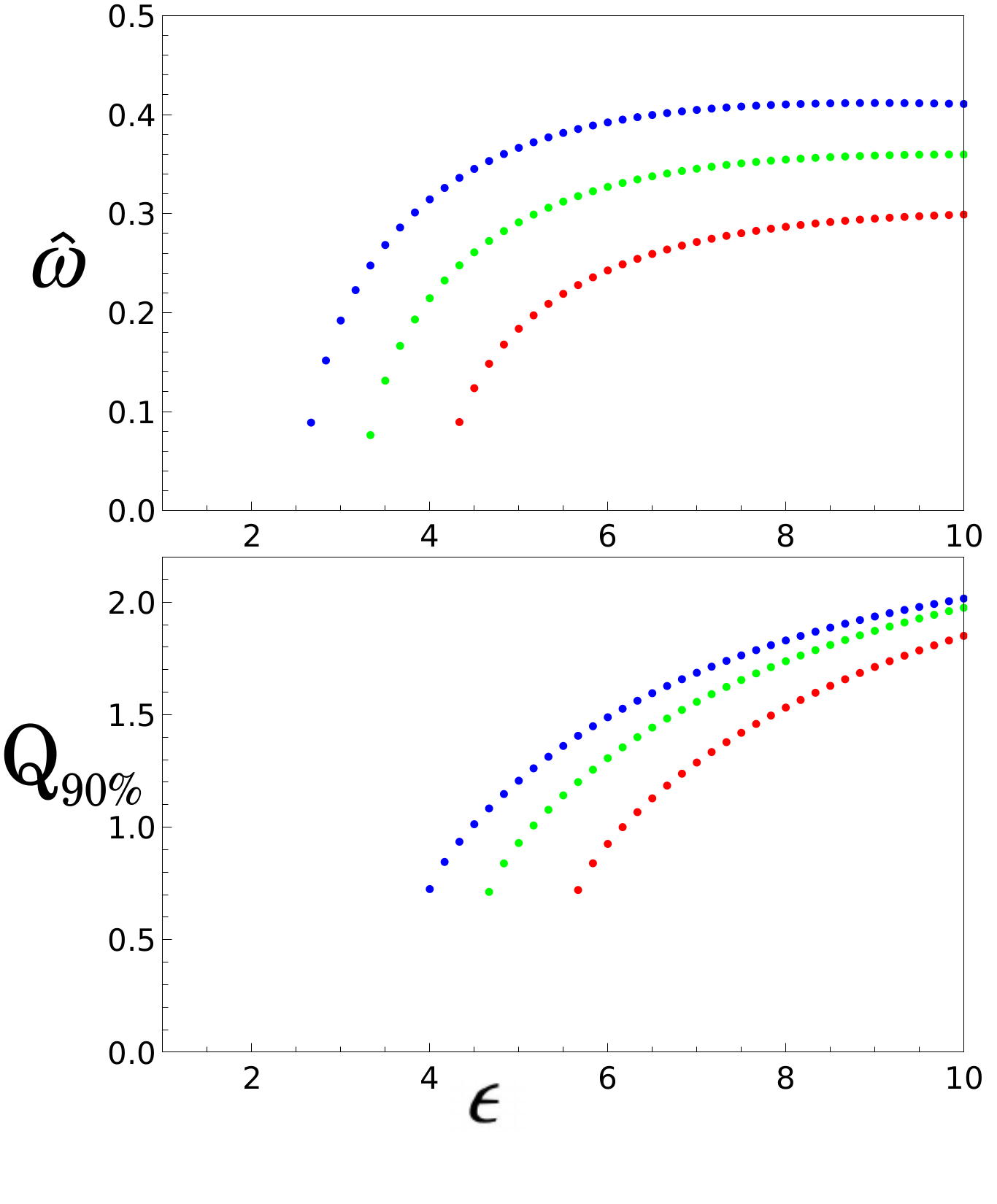

Supplement: S1 Fig — Peak frequency ω^ (top panel) and the quality factor Q90% (bottom panel) as a function of ϵ, for three value of λ: 0.75 (red), 1.0 (green) and 1.25 (blue). (TIF) [file pone.0151086.s001.tif]
